# Delirium Induced by Rapid Titration of Osilodrostat in a Patient With Cushing’s Disease: A Case Report

**DOI:** 10.7759/cureus.96607

**Published:** 2025-11-11

**Authors:** Toshiyuki Ishikawa, Ryo Sawagashira, Haruna Ito, Hiraku Kameda, Takahiro A Kato

**Affiliations:** 1 Department of Psychiatry, Tokyo Metropolitan Matsuzawa Hospital, Tokyo, JPN; 2 Department of Psychiatry, Hokkaido University, Sapporo, JPN; 3 Health Care Center, Hokkaido University, Sapporo, JPN; 4 Department of Rheumatology, Endocrinology and Nephrology, Hokkaido University, Sapporo, JPN

**Keywords:** adrenal insufficiency, cushing’s disease, delirium, depression, osilodrostat

## Abstract

Cushing’s disease frequently presents with psychiatric symptoms such as depression, anxiety, and cognitive impairment. Osilodrostat, an 11β-hydroxylase inhibitor, is used for persistent or recurrent cases, but rapid titration may precipitate adrenal insufficiency and psychiatric complications.

We report a woman in her early 40s with a history of major depressive disorder treated with clomipramine. After transsphenoidal surgery for Cushing’s disease, she remained hypercortisolemic, and hydrocortisone replacement was continued postoperatively for safety due to unstable cortisol secretion. Cortisol secretion was unstable, with day-to-day fluctuations. Osilodrostat was initiated at 2 mg/day. Shortly thereafter, urinary free cortisol (UFC) increased, and between days 3 and 5, she developed depressive symptoms, depersonalization, and suicidal ideation. These were judged to be related to cortisol elevation, and osilodrostat was rapidly titrated, reaching 40 mg/day by day 9. Depressive symptoms improved as UFC decreased. However, from day 9, she developed delirium with fluctuating consciousness, disorientation, purposeless hyperactivity, and stereotyped speech, peaking on days 10-12. During this period, blood pressure decreased, accompanied by tachycardia and fever. Infection and metabolic abnormalities were clinically excluded. Symptoms resolved spontaneously by day 14, with amnesia for the episode, and she was discharged on day 20 without recurrence.

This case illustrates a rare clinical course where depressive symptoms during cortisol elevation and delirium during cortisol reduction occurred sequentially in the same patient following rapid osilodrostat titration. The episode suggests that abrupt cortisol fluctuations may induce psychiatric symptoms even under hydrocortisone supplementation. Clinicians should avoid rapid titration and ensure close collaboration between endocrinology and psychiatry when psychiatric symptoms arise during treatment.

## Introduction

Cushing’s disease is caused by an adrenocorticotropic hormone (ACTH) secreting pituitary adenoma, leading to chronic hypercortisolism. In addition to physical features such as central obesity, moon face, and hypertension, psychiatric symptoms including depression, anxiety, and cognitive impairment are frequently observed [1-3]. Depression occurs in 40-60% of patients and is associated with increased suicide risk. Anxiety and cognitive impairment are also common, and psychiatric symptoms may even precede the physical manifestations. Thus, psychiatrists may encounter such patients at an early stage, and it is clinically important to consider underlying endocrine disorders [1,3]. The first-line treatment is transsphenoidal surgery, but remission is not always achieved [4].

Osilodrostat, an oral 11β-hydroxylase inhibitor, is primarily used for the treatment of persistent or recurrent Cushing’s disease. By inhibiting cortisol synthesis, it effectively lowers circulating cortisol levels, thereby improving the clinical manifestations of hypercortisolism. The phase III LINC 3 trial demonstrated its efficacy [5], but adverse events such as adrenal insufficiency and psychiatric symptoms have been reported [6-8]. Acute adrenal insufficiency can present with hypotension, tachycardia, fever, and gastrointestinal symptoms, and in severe cases with impaired consciousness or delirium [9]. To minimize these risks, gradual titration in 2-mg increments at intervals of at least two weeks is recommended [6].

For monitoring treatment efficacy, urinary free cortisol (UFC) is widely used as a reliable marker reflecting total cortisol secretion over 24 hours and serves as a standard index of disease activity and treatment response [1,2].

## Case presentation

The patient was a 43-year-old woman with a history of major depressive disorder since her early twenties, treated mainly with clomipramine. Although she experienced recurrent episodes, she was able to continue working as a clinical psychologist, with occasional sick leave. Her past history included papillary thyroid carcinoma treated surgically, followed by hypothyroidism managed with levothyroxine 75 µg/day.

In her thirties, she developed treatment-resistant hypertension. In March 2024, inferior petrosal sinus sampling confirmed Cushing’s disease. In April 2024, she underwent transsphenoidal surgery and started hydrocortisone replacement at 30 mg/day. However, hypercortisolism and elevated ACTH persisted. Cortisol levels showed marked day-to-day fluctuations rather than being consistently elevated, and replacement therapy was continued for safety.

In June 2024, she was admitted to our endocrinology department because of persistent disease activity. Psychiatry was consulted due to her psychiatric history. At admission, she was alert, cooperative, and exhibited neither depressive nor psychotic symptoms. Clomipramine was continued. Physical examination revealed a BMI of 27.5, central obesity, moon face, and violaceous striae. Blood pressure was 155/105 mmHg. Routine chemistry and thyroid function were within normal limits. Endocrinological work-up confirmed persistent hypercortisolism: the 24-hour UFC was markedly elevated (409.2 µg/day; normal < 50 µg/day), midnight serum cortisol was inappropriately high (14.3 µg/dL; normally suppressed at night), and dexamethasone suppression testing failed to suppress morning cortisol (9.7 µg/dL after 0.5 mg dexamethasone). Corticotropin-releasing hormone stimulation testing demonstrated an exaggerated ACTH response (63.6 → 105.0 pg/mL), consistent with pituitary-dependent Cushing’s disease. Postoperative brain MRI showed only expected surgical changes without new lesions.

**Figure 1:**
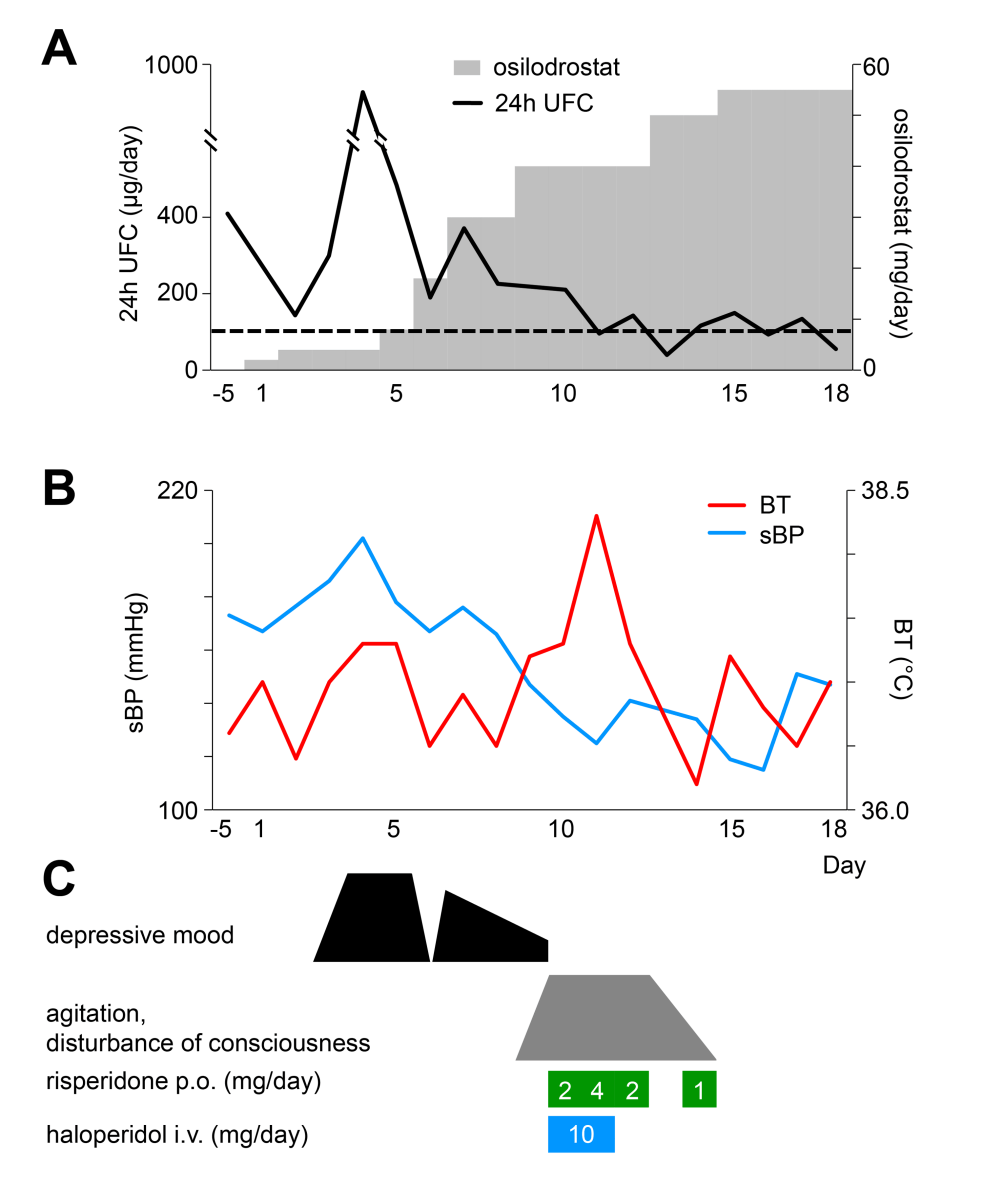
Timeline of clinical events and interventions in the present case. Panel (A) shows the osilodrostat dosage and 24-hour urinary free cortisol (UFC) levels; panel (B) depicts vital signs (sBP, systolic blood pressure; BT, body temperature); and panel (C) illustrates psychiatric symptoms and the dosages of antipsychotic medications, all plotted against treatment days.

Figure 1 illustrates the clinical course in this case. Osilodrostat was initiated at 2 mg/day on day 1. UFC unexpectedly rose thereafter, and between days 3 and 5, she developed depressed mood, depersonalization, and suicidal ideation. These psychiatric symptoms were judged to be associated with increased cortisol secretion. Antidepressant adjustment was not attempted. Instead, priority was given to endocrine control, and osilodrostat was rapidly up-titrated. Although the risk of adrenal insufficiency was considered, treatment was deemed safe under hydrocortisone supplementation. By day 9, the dose of osilodrostat reached 40 mg/day, UFC decreased, and depressive symptoms improved.

However, from day 9 onward, delirium and psychomotor agitation emerged, peaking on days 10-12. She displayed fluctuating consciousness, global disorientation, impaired attention, purposeless hyperactivity, stereotyped behaviors, and repetitive utterances of meaningless numbers. She wandered barefoot and occasionally shouted fragmented phrases such as “Say you love me.” Anxiety and insomnia were prominent, but hallucinations and self-disturbances were absent.

At that time, her vital signs showed a decline in blood pressure from 155/105 mmHg to 125/59 mmHg, a pulse rate of 110/min, and a temperature of 38.3°C. Electrolytes and glucose were normal, and no inflammatory response or other signs of infection were detected. Because of marked psychomotor agitation, imaging and EEG were not performed. Risperidone and haloperidol were given but were ineffective.

At onset, the delirium was interpreted as a manifestation of hypercortisolism, partly because it occurred during a holiday when comprehensive evaluation was not feasible. Osilodrostat was therefore not reduced. As her symptoms improved spontaneously and she remained stable under hydrocortisone supplementation, the dose was maintained. Since the delirium resolved completely and did not recur, additional imaging or EEG was not performed.

By day 14, delirium had resolved, and the patient reported amnesia for the episode. No recurrence occurred, and she was discharged on day 20 at her and her family’s request. Outpatient follow-up confirmed stable status without recurrence of delirium. 

## Discussion

This case illustrates an unusual clinical course in which qualitatively distinct psychiatric symptoms appeared sequentially during rapid titration of osilodrostat. The initial depressive phase coincided with a transient rise in UFC and may have been related to unstable cortisol secretion that had already been observed prior to admission. Although not sufficient for a formal diagnosis, such variability is reminiscent of cyclical Cushing’s disease [10], which has also been associated with mood fluctuations [1,3]. Previous studies have demonstrated the link between hypercortisolism and depression [1,3], and our case is consistent with these findings during the early phase of treatment.

In contrast, the subsequent delirium phase was accompanied by hypotension, tachycardia, and fever, resembling adrenal insufficiency. Similar neuropsychiatric manifestations, including delirium and psychosis, have been described in previous reports of adrenal insufficiency [8,9]. However, unlike those cases, where symptoms typically emerged after drug withdrawal, delirium in our patient developed rapidly following dose escalation. This temporal pattern suggests that even transient cortisol reductions may precipitate acute neuropsychiatric symptoms.

Taken together, these observations both align with and extend prior findings linking cortisol dysregulation to psychiatric manifestations. Our case supports previous evidence that hypercortisolism is associated with depressive symptoms [1,3], whereas hypocortisolism predisposes to delirium or psychosis [8,9]. Importantly, it also highlights a dynamic aspect of this relationship: abrupt cortisol fluctuations themselves, regardless of direction, may transiently disrupt neuroendocrine homeostasis and trigger psychiatric symptoms. This interpretation is consistent with reports of cyclical Cushing’s disease showing alternating mood states [10], but it differs in that the fluctuation here was iatrogenic and temporally linked to rapid pharmacologic titration.

Pharmacological factors may have further amplified these effects. Clomipramine and antipsychotics such as haloperidol and risperidone are known to cause confusion or agitation, particularly under hormonal stress. It is therefore plausible that psychotropic drug interactions and cortisol fluctuations acted synergistically to produce the observed neuropsychiatric manifestations.

This report has several limitations. The onset of delirium occurred during a holiday, and severe agitation precluded blood sampling for serum cortisol, ACTH testing, or therapeutic steroid administration. Thus, strict diagnostic criteria for adrenal insufficiency could not be fulfilled. Nonetheless, the clinical presentation, with hypotension, tachycardia, fever, and altered consciousness, was consistent with an adrenal insufficiency-like state. Electrolytes, glucose, and inflammatory markers remained within normal limits, making infection or metabolic causes unlikely. However, structural or neurological contributors could not be completely excluded because imaging and EEG were not performed. Although the clinical picture resembled adrenal insufficiency, true adrenal crisis was unlikely given the normal electrolyte levels, spontaneous recovery, and maintained oral intake. Therefore, this episode may be better characterized as a state of functional adrenal dysregulation rather than frank adrenal insufficiency.

## Conclusions

This case highlights a rare course in which depressive symptoms during cortisol elevation and delirium during cortisol reduction occurred sequentially in the same patient following rapid titration of osilodrostat. The episode suggests that even under hydrocortisone supplementation, abrupt cortisol fluctuations can induce psychiatric symptoms. However, because some observations were paradoxical and certain assessments could not be performed during the acute phase, these interpretations should be made with caution. The episode may represent a state of functional adrenal dysregulation rather than distinct phases of hyper- or hypocortisolism.

This case offers two clinical lessons. First, osilodrostat should be titrated gradually according to established guidelines. Second, if psychiatric symptoms arise during treatment, they are best managed through close collaboration between endocrinology and psychiatry.
